# Contrast-Enhanced Transcranial Doppler for Detecting Residual Leaks—A Single-Center Study on the Effectiveness of Percutaneous PFO Closure

**DOI:** 10.3390/jcm14186483

**Published:** 2025-09-15

**Authors:** Malwina Smolarek-Nicpoń, Grzegorz Smolka, Aleksandra Michalewska-Włudarczyk, Piotr Pysz, Anetta Lasek-Bal, Wojciech Wojakowski, Andrzej Kułach

**Affiliations:** 1 Department of Cardiology, School of Health Sciences in Katowice, Medical University of Silesia, 40-752 Katowice, Poland; 2 Third Department of Cardiology, School of Medicine in Katowice, Medical University of Silesia, 40-752 Katowice, Poland; 3 Department of Neurology, School of Health Sciences, Medical University of Silesia in Katowice, 40-752 Katowice, Poland; 4 Department of Neurology, Upper-Silesian Medical Centre, Medical University of Silesia in Katowice, 40-752 Katowice, Poland

**Keywords:** patent foramen ovale closure, percutaneous closure, residual leaks, contrast-enhanced transcranial Doppler

## Abstract

**Background:** A persistent connection between the atria, known as a patent foramen ovale (PFO), is present in approximately 25% of the general population. PFO closure is indicated in patients under 60 years of age who have experienced an embolic stroke of undetermined source (ESUS) or transient ischemic attack (TIA) confirmed by neurological imaging, and in selected cases of peripheral embolism. Follow-up after the procedure is indicated to confirm the position of the occluder, assess the effectiveness of the closure, and evaluate any potential thrombus formation on the device. **Methods:** We analyzed data from 75 consecutive patients who underwent percutaneous PFO closure procedures and were followed up for at least one year. The procedure was performed under fluoroscopy and transesophageal echocardiography (TEE) guidance, and occluder size selection was made using TEE multiplanar imaging (MPR). All patients had standard transthoracic echocardiography (TTE) at 1 and 6–12 months after the procedure. To assess the long-term efficacy, contrast-enhanced transcranial Doppler (ce-TCD) was performed at 12 months to record high-intensity transient signals (HITSs). Cases with positive ce-TCD had TEE performed. **Results:** During follow-up evaluations after 1 and 6–12 months (TTE), we did not observe any device dislodgements, thrombi, or residual leaks visible in TTE. ce-TCD detected HITSs in eight patients, prompting additional TEE examinations performed in seven cases. In five out of seven patients, a leak around the occluder was identified, including two patients with grade 2 HITSs. **Conclusions:** Assessing the effectiveness of PFO occluder placement is crucial for the residual embolic risk and thus the necessity of antithrombotic therapy. Even low grades of HITSs observed in ce-TCD help to identify patients with residual leaks confirmed in TEE.

## 1. Introduction

A patent foramen ovale (PFO) is present in about 25% of the general population [1]. Previous studies, including Patent Foramen Ovale Closure or Anticoagulants versus Antiplatelet Therapy to Prevent Stroke Recurrence (CLOSE), the Randomized Evaluation of Recurrent Stroke Comparing PFO Closure to Established Current Standard of Care Treatment (RESPECT), and the Device Closure Versus Medical Therapy for Cryptogenic Stroke Patients With High-Risk Patent Foramen Ovale (DEFENSE-PFO), indicate that PFO closure reduces the number of recurrent strokes compared to medical therapy [2,3].

Indications for PFO closure have been precisely outlined in the consensus document published by the Association of Cardiovascular Interventions and the Section of Grown-up Congenital Heart Disease of the Polish Cardiac Society in 2019 [4]. The procedure of PFO closure should be considered in patients under 60 years of age who have experienced an embolic stroke of undetermined source (ESUS) or transient ischemic attack (TIA) confirmed by neurological imaging, or in selected cases of peripheral embolism.

The crucial endpoint for the PFO occluder implantation is a complete sealing of the PFO channel with no residual shunt. Residual leak around the occluder is estimated to affect between 3.9% [5] and as much as 25% [6] of the population after PFO closure, depending on the diagnostic method employed. The presence of a residual right-to-left shunt (RLS) is important, as it necessitates decisions regarding the continuation of antiplatelet therapy and may warrant considerations of additional procedures to achieve full closure of the shunt.

The sensitivity and specificity of individual methods for assessing RLS vary. Contrast-enhanced transcranial Doppler (ce-TCD) offers the highest sensitivity (97%) and specificity (93%) in detecting right-to-left shunts [7]. As a positive ce-TCD does not provide a location of the shunt, it should be followed by contrast-enhanced cardiac imaging to localize a leak. Transthoracic echo (TTE) has low sensitivity, but a 96.9% specificity in detecting RLS, while Transesophageal echo (TEE) helps localize a shunt with 63.4% sensitivity and 83% [8]. While the combination of ce-TCD and TEE seems to be the strategy of choice after PFO closure, it is limited by the nature of the examinations—they are time-consuming, hard to apply as an everyday routine, and in the case of TEE, cause discomfort for patients.

The objective of this study is to evaluate the long-term efficacy of PFO occluder implantation using contrast-enhanced transcranial Doppler (ce-TCD) performed after 12 months, following TEE in cases with positive results in ce-TCD.

## 2. Materials and Methods

We retrospectively analyzed data from 75 consecutive patients who underwent percutaneous PFO closure between 2020 and 2023 and were followed up for at least one year.

### 2.1. Qualification for PFO Closure

The indications for PFO closure were confirmed by the NeuroHeart Team, following a detailed analysis that included RoPE score (Risk of Paradoxical Embolism score) and PASCAL classification [9,10].

The RoPE score is used to estimate the likelihood that a stroke in a PFO patient is attributable to the PFO and is based on clinical and imaging features, including age, hypertension, diabetes, smoking status, prior stroke or TIA, and cortical infarct on imaging.

Moreover, on a preprocedural TEE, patients were qualified as high-risk PFO (presence of atrial septal aneurysm or large shunt: more than 20 bubbles on TEE) or low-risk.

The PASCAL classification (PFO-Associated Stroke Causal Likelihood) integrates RoPE score and anatomical features of PFO (including septal aneurysm, significant shunt defined as more than 20 bubbles in the left atrium on contrast study). Based on the RoPE score, the anatomical features of the PFO patients were classified as probable, possible, or unlikely to have PFO-related stroke.

### 2.2. PFO Closure Procedure

The procedure was performed under conscious sedation and was guided by fluoroscopy and transesophageal echocardiography (TEE). To achieve homogeneity within the study group, only procedures where the Amplatzer Septal Occluder (ASO-PFO, Abbott, IL, USA) was used for PFO closure were included in the analysis.

### 2.3. PFO Measurements and Occluder Sizing

The essential dimensions comprise the length and the height (typically visualized in a slightly modified mid-oesophageal short-axis view—ca 40–60 degrees) as well as the width (visualized in the perpendicular plane, typically corresponding to a slightly modified bicaval view). We performed the measurements intraprocedurally, having a stiff wire or a delivery sheath introduced across the PFO and positioned in the left upper pulmonary vein. This ensured maximal opening of the tunnel and enabled determining the truly largest dimension. The length, height, and width of the PFO canal were measured using multiplanar reconstructions based on transoesophageal 3D TEE data. First, the position of three perpendicular planes needs to be adjusted in the acquired zoomed 3D volume containing the PFO. Two of the planes are oriented along the PFO tunnel, after which the third one finds itself exactly perpendicular to the tunnel and showing its true cross-sectional dimensions. This precision of plane adjustment is unique for 3D MPR and superior to both 2D and biplanar imaging, where the orientation of planes of measurements is much less verifiable. Furthermore, in cases of a long PFO tunnel, the 3D MPR method allows for identifying the largest dimension throughout its course. Finding the largest width in flabby structures, such as PFO, is crucial to selecting a proper occluder.

As expected from the anatomical orientation of overlapping fragments of septum primum and septum secundum, which form the PFO tunnel, it is typically the width of the tunnel that represents its largest dimension and determines the occluder selection.

### 2.4. Occluder Size Selection

**Figure 1:**
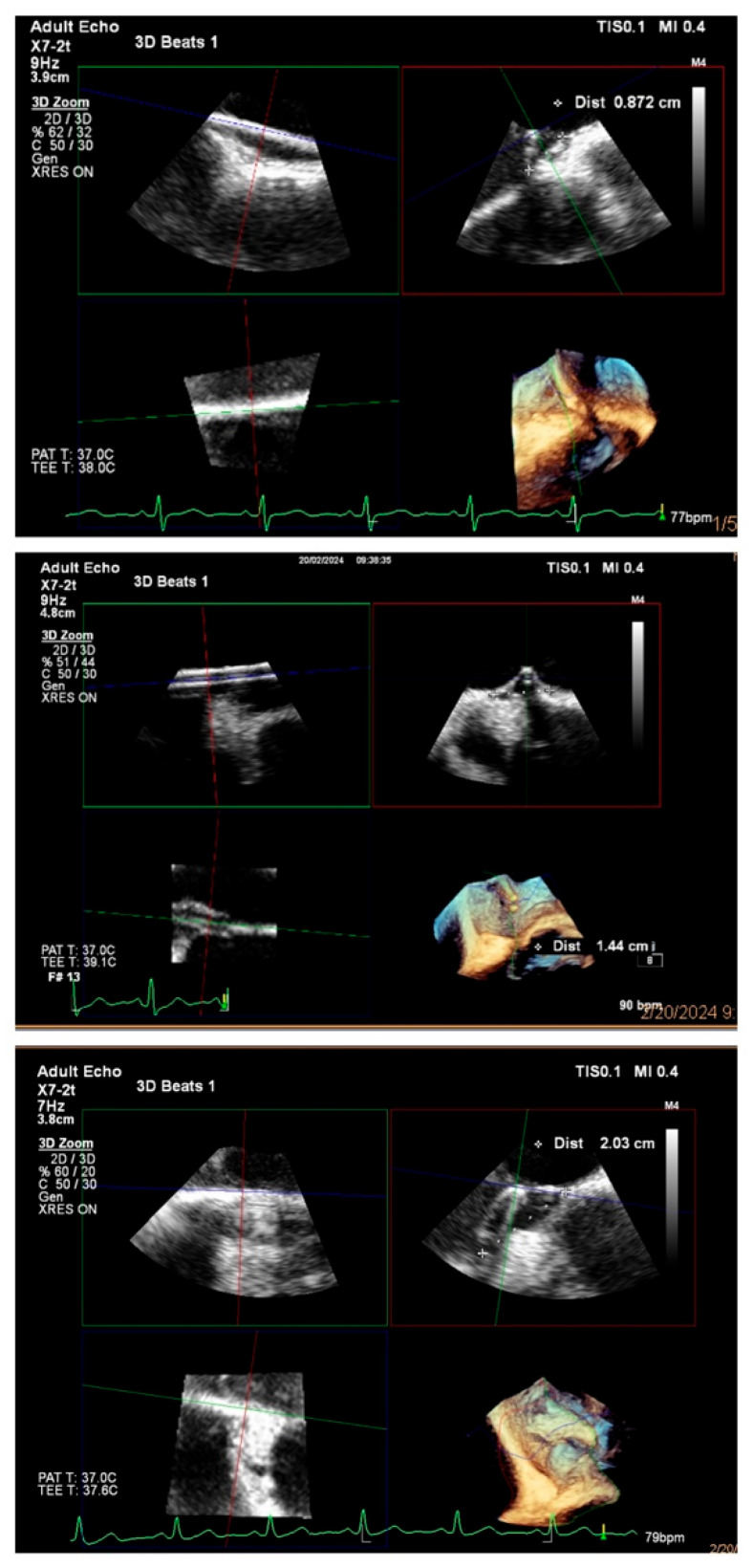
Examples of occluder selection. **Upper** panel: width < 10 mm—occluder 18 mm; **Middle** panel: width 10–15 mm—occluder 25 mm; **Lower** panel: width ≥ 15 mm and ASA—occluder 30 mm.

When selecting the appropriate occluder size, if the maximum measured width of the patent foramen ovale does not exceed 10 mm, Amplatzer PFO Occluder 18 mm is recommended (with the maximum width being smaller than the length of the disk radius). For larger PFOs, a 25 mm occluder is indicated for tunnel widths ranging from ≥10 mm to <15 mm, while a 30 mm occluder is utilized for maximum tunnel widths ≥15 mm, particularly in the presence of atrial septum aneurysm. Examples of occluder selection are given in Figure 1. Additionally, the presence of other factors, such as longer tunnel length or increased septum mobility, also influences the selection of the device size [11].

The completeness of the closure was evaluated in the final phase of the procedure using color Doppler TEE in multiple views.

### 2.5. Follow-Up

All patients had standard transthoracic echocardiography at 1 (Visit 1) and 6–12 months (Visit 2) after the procedure to assess the occluder position and the effectiveness of PFO sealing (the occurrence of any new color-Doppler signals at the level of the interatrial septum (IAS)). All patients received dual antiplatelet therapy for 6 months, which was extended in cases of residual leaks.

To assess the long-term efficacy, ce-TCD was performed 12 months after the initial treatment, and high-intensity transient signals were recorded.

With a 97% sensitivity and 93% specificity in detecting right-to-left shunts [7], this technique is utilized to identify cerebral HITS in the middle cerebral artery (MCA) during the Valsalva maneuver following intravenous contrast administration.

**Figure 2:**
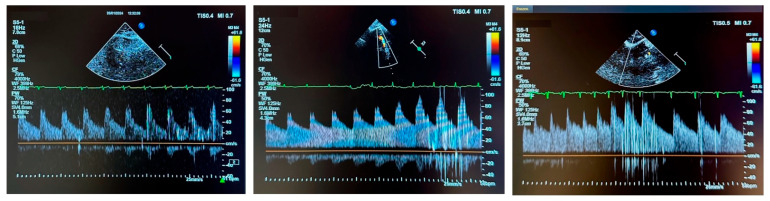
Examples of contrast-enhanced transcranial Doppler. **Left** Panel: ICC Category 2, **Middle** Panel: Category 3, **Right** Panel: Category 4.

A 2 MHz sector transducer with color Doppler capability was used to locate the MCA at a depth of 3.5 to 4 cm from the skin, within the bone window between the lateral margin of the orbit and the ear, above the zygomatic arch. After identifying the appropriate location for the transducer, a contrast mixture (10 mL total, consisting of 8 mL 0.9% NaCl, 1 mL of the patient’s blood, and 1 mL of air) was prepared using 2 syringes connected to a 3-way stopcock. The patient was then instructed to perform a Valsalva maneuver [12]. The contrast was administered during the straining phase, 8 s after the onset of the Valsalva maneuver. The number of early HITS detected served as an indicator of residual leaks, and were classified according to ICC [12,13] in 4 categories: 1: No HITS (negative; no shunt), 2: 1–10 HITS (small shunt), 3: 11–25 HITS (medium shunt) and 4: >25 HITS, including the curtain feature (large shunt). Examples of ce-TCD are shown in Figure 2.

Any category of HITSs recorded in ce-TCD prompted a full contrast-enhanced TEE examination.

All ultrasound procedures were conducted using Philips Affiniti CVs (Koninklijke Philips N.V., Amsterdam, the Netherlands). For TTE and TCD examinations, a sector transducer S5-1 with CD modality was utilized. In the case of the TEE examination, the transesophageal X7-2T transducer was used.

Data was gathered from the medical record retrospectively and de-identified before analysis. Statistical analysis was performed with MedCalc Statistical Software version 22.026 (MedCalc Software Ltd., Ostend, Belgium). The categorical variables are reported as counts and percentages. The continuous variables are presented as the mean ± SD (standard deviation) for normal distribution and the median and IQR for non-normal distribution. Normality was tested with the Shapiro–Wilk test. Differences between groups were tested using the *t*-test and the U-Mann–Whitney test. Qualitative parameters were compared using Pearson’s chi-square and McNemar’s test. The universal *p*-value level < 0.05 was considered statistically significant throughout the analyses.

This investigation was carried out in accordance with the principles outlined in the Declaration of Helsinki.

## 3. Results

The study population included 75 cases: 41 females (55%) and 34 males (45%), aged 18–77 years (median 45 years, IQR 35–53 years).

The study group’s risk profile for stroke attributable to PFO was stratified using RoPE score, PFO characteristics, and PASCAL classification. A total of 64% of participants had a RoPE score > 7. In a qualifying TEE, 51 out of 75 (68%) of patients were considered anatomically high-risk PFO (ASA or large shunt: more than 20 bubbles on TEE). In a PASCAL classification, in only two cases (3%) the relation between thrombotic complications and PFO was considered unlikely, while in 63% it was rated possible, and in 35% it was rated probable.

The most common indication for PFO closure was ischemic stroke (80%). A transient ischemic attack was an indication for closure in 12%, and in 4% it was a migraine. In three subjects (4%), peripheral embolic complications were the indication for PFO closure. In a preprocedural TEE, 51 out of 75 (68%) of patients were considered high-risk PFO (ASA or large shunt: more than 20 bubbles on TEE). Details on baseline characteristics, including PASCAL classification and RoPE score, are given in Table 1.

Based on the multiplanar reconstruction of 3D TEE, the predominant size of the Amplatzer Occluder (Abbott) implanted was 25 mm (57%) and 18 mm (35%). A size of 30 mm was the least common and accounted for 8% only. All devices were stable at the end of the procedure, and no residual leaks around the occluder were detected.

The subjects with high RoPE score (7 or more) were younger (39.3 vs. 55.1 years of age, *p* < 0.001), but there were no differences in the distribution of stroke, TIA, migraine, and positive TCD. In patients with low RoPE score, a high-risk PFO was more common than in high RoPE subjects (93% vs. 55%, *p* < 0.001).

The patients with anatomically high-risk PFO were similar in terms of age (46 ± 13 vs. 43 ± 11 years old, *p* > 0.348), sex, and occluder size distribution, while the history of stroke was more common in high-risk PFO subjects (86% vs. 66%, *p* < 0.05). TCD was positive in a similar proportion of high-risk and low-risk PFO (11% vs. 14%, *p* = 0.92).

### 3.1. 1 Month and 6–12 Months Clinical and TTE Follow-Up

During the one-month and 6–12 month follow-up visits, there were no device dislodgements recorded in TTE, and no visible (color Doppler TTE) interatrial shunt was found in any case.

### 3.2. ce-TCD and TEE Follow-Up After 12 Months

In ce-TCD, we recorded eight cases (11%) with HITS, including three in Category 4 HITS considered to be related to a large shunt, and three in Category 3 (Table 2). Out of the eight subjects, seven had a TEE performed (one patient refused). In five out of seven (6.7% of the total group), a leak around the occluder was identified. This included two patients with Category 2 HITSs, where both cases were found to be positive for shunt on TEE.

In two cases, in which TEE did not confirm a significant shunt despite positive ce-TCD, we scheduled further workup to localize the suspected shunt. One patient was lost to follow-up, while in the other one, a perfusion scintigraphy was performed, but it did not reveal any significant left-to-right shunt.

During the 12 month follow-up, no patient experienced a recurrent neurological event, and no new arrhythmia was diagnosed in any patient during the follow-up period.

## 4. Discussion

The first clinical trials using a septal umbrella date back to 1989. Back then, the imaging methods and occluder selection were different. Balloons were often used to properly size the PFO canal when used under fluoroscopy [14]. More recently, guiding of IAS occluder implantation procedures was introduced using fluoroscopy and transesophageal echocardiography, but the practice related to PFO closure remains highly center and operator-dependent. This includes intraprocedural use of TEE guidance, confirmation of the procedural success, and a strategy to select the occluder size. Moreover, the follow-up standards, although defined in position statements and guidelines, are still a subject of discussion.

### 4.1. Qualification for PFO Closure

The NeuroHeart team’s qualification of patients for PFO closure is a common practice and is being applied worldwide. The team-based approach ensures a comprehensive assessment and shared decision-making process regarding PFO closure, considering both the potential benefits and risks for the patient [15]. As mentioned, the decision is based on the anatomical features of the PFO and clinical assessment with RoPE score calculation. The results are then combined in PASCAL classification, where subjects with either high-risk PFO or RoPE score 7+ are classified as a possible relation of stroke to PFO, subjects with both (high-risk, high RoPE)-as probable, while patients with none—as unlikely. In our study, 2/3 of the subjects were classified as possible, while 1/3 were classified as probable. Patients with high vs. low RoPE scores did not differ concerning the indication for PFO closure, and the incidence of positive TCD on follow-up was similar. Interestingly, in patients with low RoPE score, an anatomically high-risk PFO was more common than in high RoPE subjects (93% vs. 55%, *p* < 0.001), which confirms a good selection of patients in the group, and, based on clinical characteristics, the patients are less likely to have a stroke related to PFO.

### 4.2. Procedure—Guidance, Occluder Selection, and Procedural Success Confirmation

Our study shows that TEE-guided PFO closure procedures ensure not only a better occluder size selection strategy but also a complete PFO closure and confirm the stability of the occluder. However, even in this population, a strict follow-up is necessary to seek a residual leak.

In our center, TEE is crucial during the procedure. Before releasing the occluder from the delivery system, a wiggle maneuver is carried out to assess its stability. In addition, the surface of the occluder is evaluated for thrombi, the relation to the surrounding structures is assessed, and the residual leaks are sought [4]. There are no specific data on the method of intraoperative assessment of residual leaks. Available sources state that CD-TEE or bubble contrast is used as a method of leak assessment [11]. Other authors, however, do not recommend administering bubble contrast at the end of the procedure [16]. We use CD-TEE and 3D-TEE to check for the completeness of the PFO closure.

Wahl et al. describe that 5% of the 620 patients enrolled in the study had a suboptimal position of the occluder (referred to as the Packman sign), which was associated with an intraoperative change in its size [17]. In our study, an MPR TEE method for occluder sizing allows for better size selection, thus reducing the suboptimal device placement.

Choosing the right size of the occluder for the PFO canal is crucial to achieving complete closure. The method described by Demulier et al. [18], using 3D TEE imaging, has been used for many years at the local center. In addition, multiplanar imaging (MPR TEE) is used to precisely determine the site of the largest PFO width, which allows the largest PFO opening width to be found. Based on this dimension, the occluder is selected.

In patients included in this registry, we used Amplazer PFO Occluder size 18 mm in 26 patients (35%), 25 mm—43 patients (57%), and 30 mm—6 patients (8%). Smaller occluders may promote residual leaks, TIA recurrence, and device embolization [6,19], but on the other hand, larger ones are less likely to adhere to IAS and may be linked to a longer endothelialization time [20], which is necessary to obtain complete closure of the atrial communication and prevent thrombus formation [21].

### 4.3. Follow-Up, Long-Term Effectiveness of PFO Closure

The evaluation of the long-term effectiveness of PFO closure and exclusion of residual leaks that may cause recurrent neurological events is not precisely defined. The position paper released in 2019 suggests (1) a TTE before hospital discharge, (2) ce-TCD at least once beyond six months to assess effective PFO closure and thereafter, if residual shunt persists, and (3) c-TEE or c-TTE in case of large residual shunt at ce-TCD, or recurrent events, or symptoms during follow-up [22].

Yared K. et al. followed up using TTE with CD function or with bubble saline at rest and during the Valsalva trial. TEE was used only in the case of subsequent neurological events [23]. Vitarelli et al. routinely performed TTE control at 1 day and 1 month post-treatment, and TEE at 6 months. If no residual leakage was visible, another TEE test to confirm the final seal of the PFO was performed after another 6 months. If residual leakage was visible, TEE was repeated every 6 months until the leak was confirmed to be sealed two times [24].

We performed clinical and echocardiographic (TTE) examinations at 1 and 6–12 months after transcatheter PFO closure. The process of endothelialization of the device took 6–12 months. Most residual leaks tended to close spontaneously within 12 months after the procedure [25]. Therefore, the final leak check with ce-TCD was set at least 12 months after the procedure.

ce-TCD, as mentioned, has a high sensitivity in detecting residual shunts. In the majority of reports, however, the threshold for a significant leak is set to >10 HITS or even more than 20 HITSs. Our study showed that even in the lowest category (HITSs 1–10), significant shunts may be visualized in the TEE. Moreover, there are subjects where positive TCD is not associated with a visible shunt in TEE, which warrants a further workup for extracardiac shunts. Low-grade HITS on ce-TCD (or even false negative ce-TCD) can happen in patients with clinically significant PFOs and are commonly related to technical issues. These include improper Valsalva, the wrong timing of signal acquisition, poor quality of microbubble contrast, or poor quality of imaging. In our center, every effort is made to rule out the technical factors. Yet, there are still physiological (low right atrial pressure, hypovolemia) and anatomical reasons (e.g., Persistent Eustachian valve [26]) when a significant leak in color Doppler may be associated with low-grade HITS.

The reasons for positive ce-TCD with negative TEE include extracardiac shunts, pulmonary arteriovenous malformations, and these have to be confirmed or excluded in a dedicated study, including scintigraphy, as we did in one case. The more common reasons for false positive ce-TCD are, however, the technical issues. These include inadequate Valsalva maneuver, inadequate contrast injection, and finally, technical issues related to equipment and software used [27,28] as well as operator misinterpretation, i.e., wrong distinction between early (cardiac) and late (pulmonary) bubbles.

The false positive ce-TCDs are, in most cases, the low-grade results (1–10 HINTS). Our study shows that even in this group, it is worth verifying the result in TEE.

Although the false positive ce-TCD results may lead to extensive workup in search for intra- and extracardiac shunt, it rarely leads to intervention if the shunt is not confirmed in TEE.

Although the presence of the residual shunt is related to the risk of stroke, the recent data show that the size of the shunt matters [29]. Moreover, even properly selected patients who had PFO closure have a 2% 4-year risk of stroke, which is six-fold higher than the general population, as Bonnesen et al. report [30]. In our study, the prevalence of residual shunt was low, and no strokes were observed during follow-up; however, the study was not powered to detect clinical endpoints.

### 4.4. Study Limitations

This study has several limitations. Firstly, the number of participants is relatively low, and the study is not powered to assess clinical safety (i.e., device dislodgement rate, procedural complications) and clinical efficacy (expressed as long-term stroke rate) of the procedure performed in the described way. However, the study aimed to show the long-term efficacy when a combination of highly sensitive and highly specific modalities is used. Moreover, we show that even in the settings of a 100% procedural success and no major findings on regular follow-up, in almost 7%, the shunt is detectable after a year. Secondly, due to a low number of clinical details and a low number of detected shunts, an extensive statistical analysis (including multiple regression analysis) was not possible to identify the factors related to the shunts detected after a year. Thirdly, we present the results in a cohort of PFO closure with a specific sizing method and follow-up with a specific regimen of residual leak screening and confirmation examination, which limits generalizability. Moreover, this study does not provide a control group, either in terms of the occluder selection or in terms of an alternative strategy to seek residual leaks. Further research is necessary to address these issues. Finally, TEE was performed only in subjects with positive ce-TCD. Therefore, the investigation cannot conclude if there were patients with normal TCD who had undetected leaks.

### 4.5. Clinical Implications

The major implication of this study is that PFO closure is a procedure that requires a detailed and long-term follow-up, even if the procedural success is high, and the clinical post-procedural follow-up is uneventful. A screening with ce-TCD-a, a relatively easy, inexpensive, and highly sensitive tool, allows for selecting a group of patients with residual shunt, which prompts extended antiplatelet therapy and allows for planning further procedural steps in selected cases.

## 5. Conclusions

Evaluating the efficacy of PFO occluder placement is crucial for the residual embolic risk and thus the necessity of antithrombotic therapy. Even low-grade HITS detected on ce-TCD can identify patients with residual leaks confirmed in TEE. Assessing the completeness of the PFO closure with ce-TCD may indicate patients in whom DAPT extension should be considered, along with further echocardiographic and ce-TCD follow-up beyond 12 months, and reconsideration for residual leak closure should be indicated.

Patient Consent: The authors confirm that patient consent is not applicable to this article. This is a retrospective report using de-identified data; therefore, the IRB declared that the patient’s consent is not required in this study.

## Figures and Tables

**Table 1:** Baseline characteristics of the study groups.

	Total n
Sex (Female) n, n%	41 (55%)
Age	
18–30	8 (11%)
31–40	22 (29%)
41–50	21 (28%)
>50	24 (32%)
Median (IQR)	45 (35–53)
Indication for PFO closure	
Stroke	60 (80%)
TIA	9 (12%)
Migraine	3 (4%)
other causes	3 (4%)
PASCAL	
unlikely	2 (3%)
possible	47 (63%)
probably	26 (35%)
RoPE	
<7	27 (36%)
≥7	48 (64%)
Occluder size	
18 mm	26 (35%)
25 mm	43 (57%)
30 mm	6 (8%)

PFO, patent foramen ovale; RoPE, Risk of Paradoxical Embolism score; IQR, interquartile range.

**Table 2:** Contrast-enhanced TCD findings and TEE verification.

	No of ce-TCD (+) Cases	Shunt Confirmed in TEE
Category 4 HITS (>25)	3	2 positive, 1 negative
Category 3 HITS (11–25)	3	1 positive, 1 negative, 1 LTFU
Category 2 HITS (1–10)	2	2 Positive
Total ce-TCD (+)	8	5

TCD, transcranial Doppler; HITS, high-intensity transient signals; LTFU, lost to follow-up; TEE, transesophageal echocardiography.

## Data Availability

The original contributions presented in this study are included in the article. Further inquiries can be directed to the corresponding author.
